# Supplementary material for: Tandem Transesterification–Esterification Reactions Using a Hydrophilic Sulfonated Silica Catalyst for the Synthesis of Wintergreen Oil from Acetylsalicylic Acid Promoted by Microwave Irradiation
Source: Molecules. 2022 Jul 26;27(15):4767. doi: 10.3390/molecules27154767 (PMC9330306; doi:10.3390/molecules27154767)
Supplement: Supplementary file 1 [file molecules-27-04767-s001.zip › molecules-1770301-supplementary.pdf]

Tandem transesterification-esterification reactions using a hydrophilic sulfonated silica catalyst in the synthesis of Wintergreen oil from Acetyl Salicylic Acid in a process promoted by microwave irradiation

Sandro L. Barbosa,[a*] David Lee Nelson,[a] Milton de S. Freitas,[a] Wallans Torres Pio dos Santos,[a] Stanlei I. Klein,[b] Giuliano C. Clososki,[c] Franco J. Caires,[c] Alexandre P. Wentz[d]

[a]Department of Pharmacy, Universidade Federal dos Vales do Jequitinhonha e Mucuri-UFVJM, Campus JK, Rodovia MGT 367 - Km 583, nº 5.000, Alto da Jacuba, CEP 39100-000, Diamantina/MG, Brazil. e-mail: sandro.barbosa@ufvjm.edu; dleenelson@gamil.com; freitas.milton@hotmail.com; wallanst@ufvjm.edu.br

[b]Department of General and Inorganic Chemistry, Institute of Chemistry, São Paulo State University-Unesp, R. Prof. Francisco Degni 55, Quitandinha, CEP-14.800-900 Araraquara/SP, Brazil; e-mail: stanleiklein@gmail.com

[c]Department of Physics and Chemistry, Faculdade de Ciências Farmacêuticas de Ribeirão Preto, São Paulo University-USP, Av. do Café s/n, CEP-14.040-903 Ribeirão Preto/SP, Brazil; e-mail:gclososki@yahoo.com.br; fjcaires@usp.br

[d]Centro universitário SENAI-CIMATEC, Av. Orlando Gomes, 1845, Piatã, 41650-010, Salvador, BA, Brazil; e-mail: alexandre.wentz@fieb.org.br

*Corresponding author. Tel.: +55-38-35321234; fax: +55-38-35321234; e-mail: sandro.barbosa@ufvjm.edu.br

---

Abstract: SiO_2-SO_3H, with a surface area of 115 m^2/g, pore volumes of 0.38 cm^3g^{-1} and 1.32 mmol H^+/g, was used as a 20% w/w catalyst for the preparation of methyl salicylate (Wintergreen oil - WO) from Acetyl Salicylic Acid (ASA). A conversion of 75% was achieved in a microwave reactor during 40.0 minutes at 120 ºC in MeOH. The resulting crude product, which could be purified by flash chromatography. The catalyst could be re-used three times.

Keywords: Sulfonated silica catalyst, wintergreen oil, methyl salicylate, tandem transesterification-esterification, green chemistry, methylating agent, microwave irradiation.

---

1. List of Chemicals

Reagents	CAS
Acetylsalicylic acid	50-78-2
Salicylic acid	69-72-7
Methyl salicylate	119-36-8
Methanol	67-56-1
Sodium sulfate	7757-82-6

2. NMR spectra, interpretation of ^{1}H NMR Spectrum and characterization data of MS (WO) compound.

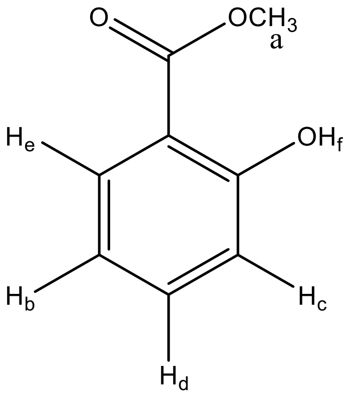

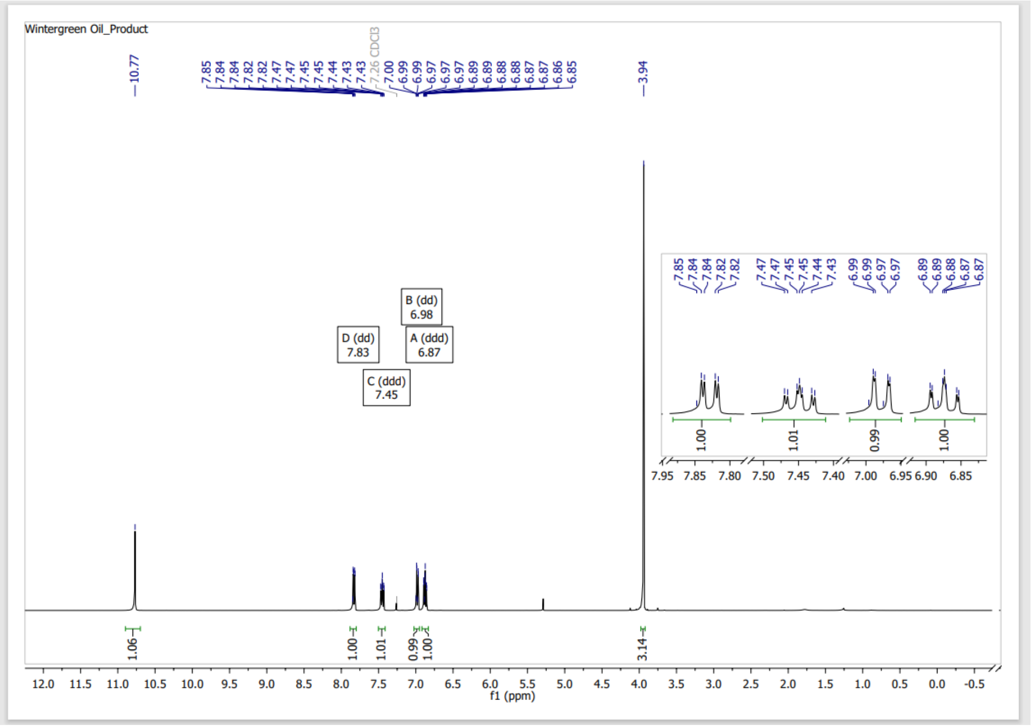

Figure S1. ^{1}H-NMR [400 MHz, $CDCl_3$, δ (ppm)] of MS: 3.94, s, 3H (CH_3); 6.88, ddd, 1H (H_b) (J_{be} = 8.0 Hz, J_{bd} = 7.1 Hz and J_{bc} = 1.1 Hz); 6.98, dd, 1H (H_c) (J_{cd} = 8.5 Hz, J_{cb} = 1.1 Hz); 7.45, ddd, 1H (H_d) (J_{dc} = 8.5 Hz, J_{db} = 7.1 Hz, J_{de} =1.7 Hz); 7.84, dd, 1H (H_e) (J_{eb} = 8.0 Hz, J_{ed} = 1.7 Hz) and 10.75, s, 1H (phenolic proton).

2.2. Interpretation of ^{1}H NMR Spectrum.

a This methyl group (δ 3.94, s, 3 H) is unsplit and deshielded by the electronegative oxygen.

b This aromatic proton (δ 6.88, ddd, 1 H) is coupled through 3 bonds with proton e (J_{be} = 8.0 Hz) and proton d (J_{bd} = 7.1 Hz) and 4 bonds with proton c (J_{bc} = 1.1 Hz).

c This aromatic proton (δ 6.98, dd, 1 H) is coupled through 3 bonds with proton d (J_{cd} = 8.5 Hz) and 4 bonds with proton b (J_{cb} = 1.1 Hz).

d This aromatic proton (δ 7.45, ddd, 1 H) is coupled through 3 bonds with proton c (J_{dc} = 8.5 Hz) and proton b (J_{db} = 7.1 Hz) and 4 bonds with proton e (J_{de} =1.7 Hz).

e This aromatic proton (δ 7.84, dd, 1 H) is coupled through 3 bonds with proton b (J_{eb} = 8.0 Hz) and 4 bonds with proton d (J_{ed} = 1.7 Hz).

f This phenolic proton (δ 10.75, s, 1 H) is unsplit and is farther downfield than is typical for other phenols. This can be attributed to the hydrogen bonding that occurs with the carbonyl oxygen of the ester.

Note the much smaller coupling constants for the long range (4-bond) couplings. The coupling constants, splitting patterns and chemical shifts can be used to match each resonance to the corresponding proton of the compound. Proton a integrates as 3H, is unsplit and modestly deshielded by the neighboring oxygen methyl group. Identifying this as the methyl group of the ester is straightforward. Proton f integrates as 1H, is unsplit and far downfield, identifying this as the phenolic hydrogen. Identifying the aromatic protons is more involved. The hydroxyl group should shield protons b and c through electron donation to its ortho and para positions. The carbonyl should deshield protons d and e through electron withdrawal from its ortho and para positions. Proton b and proton e share a 3-bond coupling constant (J_{be} = J_{eb} = 8.0 Hz) and are thus adjacent. Proton c and proton d share a 3-bond coupling constant (J_{cd} = J_{dc} = 8.5 Hz) and are thus adjacent. Proton b and proton d share a 3-bond coupling constant (Jbd = Jdb = 7.1 Hz) and are thus adjacent. The smaller 4-bond coupling constants reinforce these identifications.

Proton b and proton c share a 4-bond coupling constant (Jbc = Jcb = 1.1 Hz) and thus are meta to each other. Proton d and proton e share a 4-bond coupling constant (Jde = Jed = 1.7 Hz) and thus are meta to each other.

3. NMR spectra, interpretation of ^{13}C NMR Spectrum and characterization data of MS (WO) compound.

3.1. NMR spectra

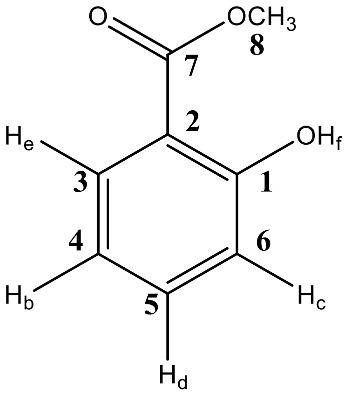

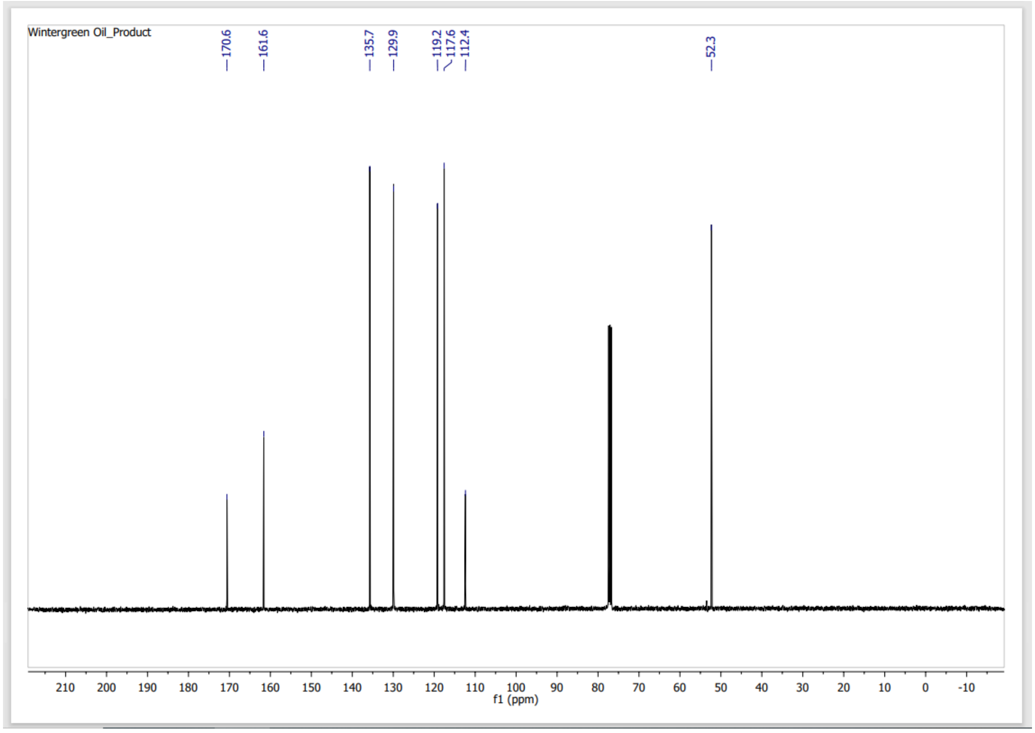

Figure S2. ^{13}C NMR [100 MHz, $CDCl_3$, δ (ppm)] of MS: 170.6 C=O or Ar quaternary, 161.6 C=O or Ar quaternary, 135.7 Ar CH, 129.9 Ar CH, 119.2 Ar CH, 117.6 Ar CH, 112.4 Ar quaternary and 52.3 CH_3.

3.2. Interpretation of ^{13}C NMR Spectrum.

Decoupled, eight peaks are observed, (ignoring the solvent peaks!). The methyl carbon is immediately recognizable due to its high field chemical shift. The carbonyl group is typically down field and could be either 170.6 or 161.6 ppm. Since quaternary carbons do not relax as quickly as tertiary, secondary and primary carbons then the peak intensities (and the chemical shifts!) indicate which peaks correspond to Ar CH and which to Ar quaternary carbons.

4. GC Chromatogram of MS compound.

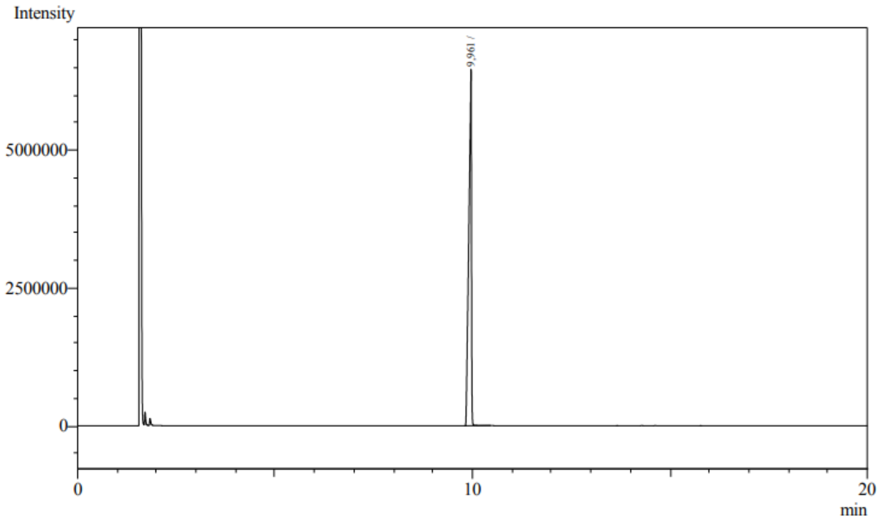

Peak#	Ret.Time	Area	Height	Conc.	Unit	Mark	ID#	Cmpd Name
1	9,961	29483915	6439642	100,000				
**Total**		29483915	6439642					

4.1. Figure S3. GC Chromatogram of MS (WO) compound.

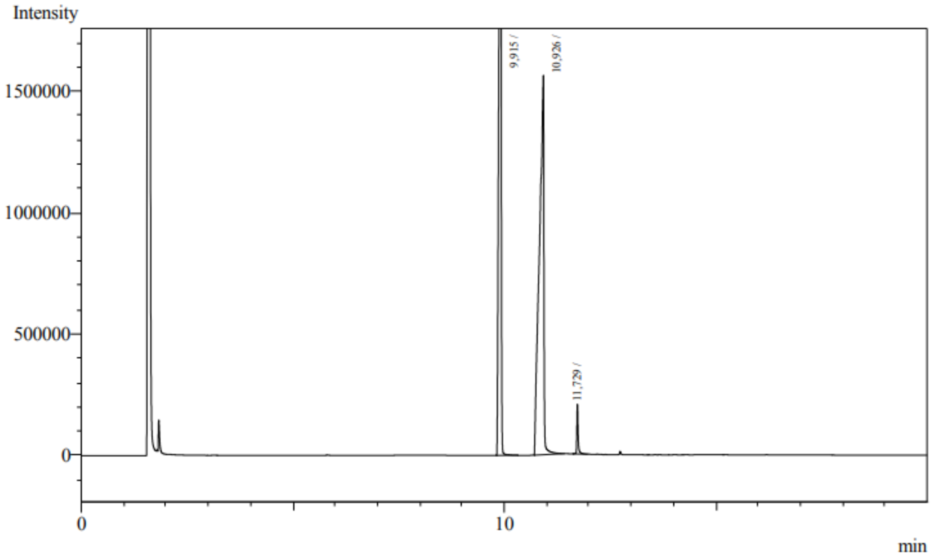

Peak#	Ret.Time	Area	Height	Conc.	Unit	Mark	ID#	Cmpd Name
1	9,915	12533432	3879347	51,245				
2	10,926	11501391	1545163	47,025				
3	11,729	423140	202022	1,730				
**Total**		24457963	5626532					

4.2. Figure S4. GC Chromatogram of MS compound. MS produced from esterification reaction: SA with MeOH, SiO_2-SO_3H 20% w/w. Chromatogram obtained after 20 min.

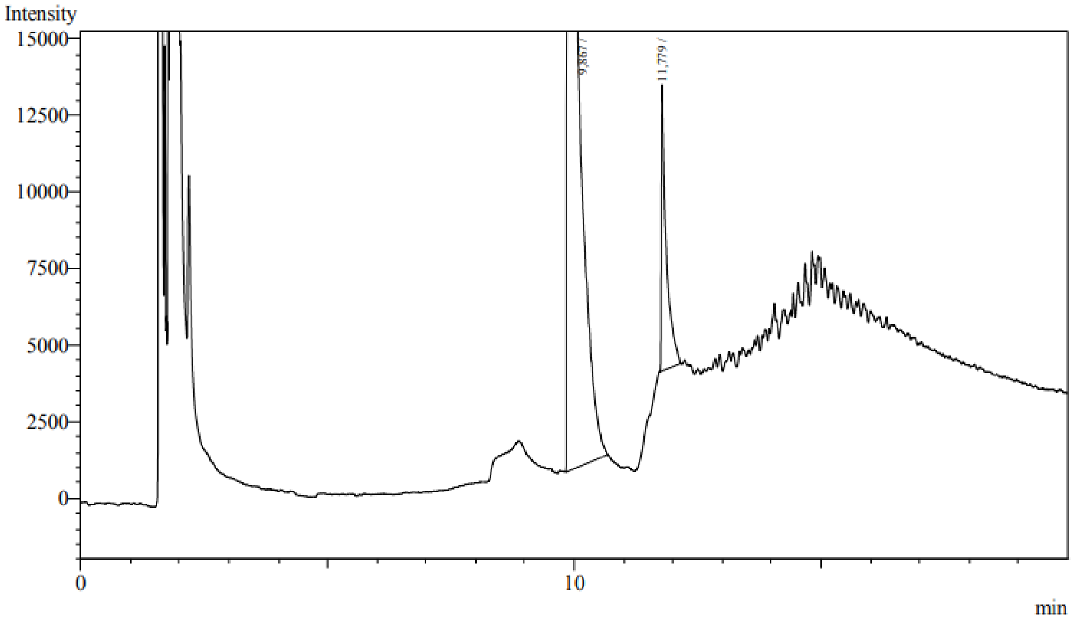

Peak#	Ret.Time	Area	Height	Conc.	Unit	Mark	ID#	Cmpd Name
1	9,867	958086	171335	93,673				
2	11,779	64717	9263	6,327				
**Total**		1022803	180598					

4.3. Figure S5. GC Chromatogram of MS compound. MS produced from esterification reaction: SA with MeOH, SiO_2-SO_3H 20% w/w. Chromatogram obtained after 40 min.

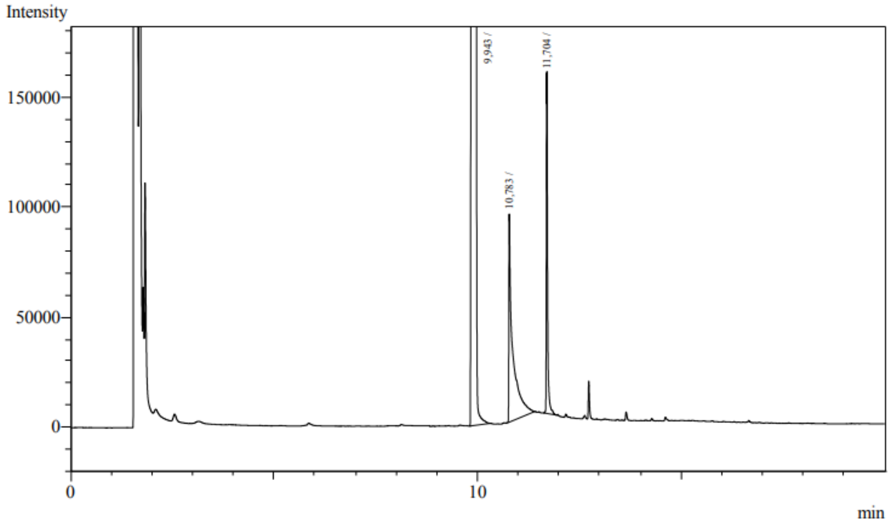

Peak#	Ret.Time	Area	Height	Conc.	Unit	Mark	ID#	Cmpd Name
1	9,943	22382151	5437818	96,022				
2	10,783	613521	94790	2,632				
3	11,704	313735	152149	1,346				
**Total**		23309407	5684757					

4.4. Figure S6. GC Chromatogram of MS compound. MS produced from transesterification and esterification reactions: ASA with MeOH, SiO_2-SO_3H 20% w/w. Chromatogram obtained after 90 min.

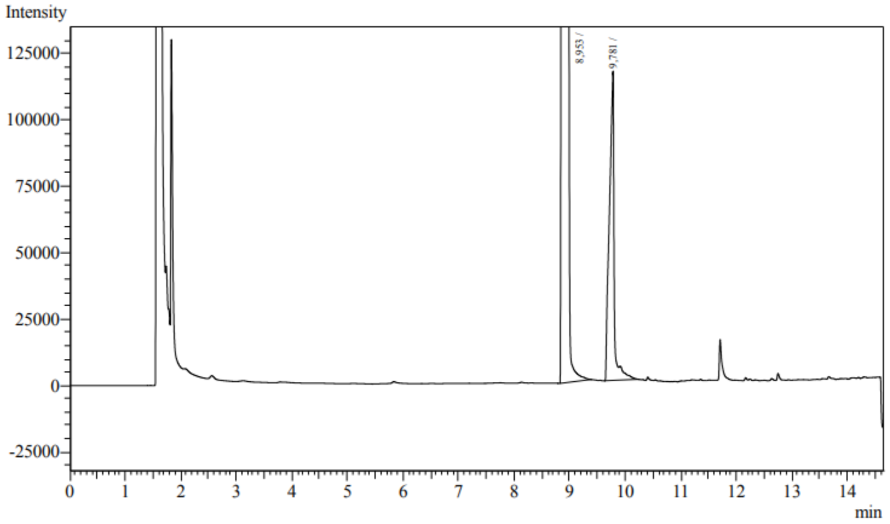

Peak#	Ret.Time	Area	Height	Conc.	Unit	Mark	ID#	Cmpd Name
1	8,953	27498529	6472486	97,549				
2	9,781	690825	115062	2,451				
**Total**		28189354	6587548					

4.5. Figure S7. GC Chromatogram of MS compound. MS produced from transesterification and esterification reactions: ASA with MeOH, SiO_2-SO_3H 20% w/w. Chromatogram obtained after 120 min.

5. NMR spectra, interpretation of ^{1}H NMR Spectrum and characterization data of Acetylsalicylic acid (ASA) compound.

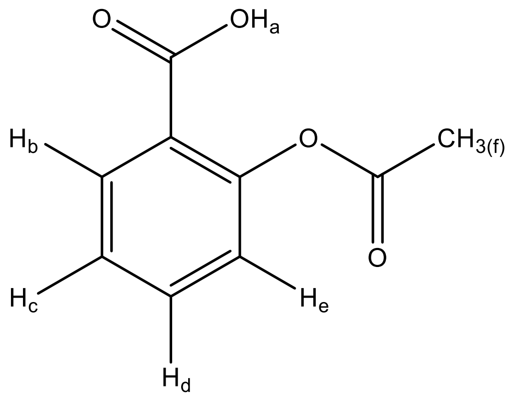

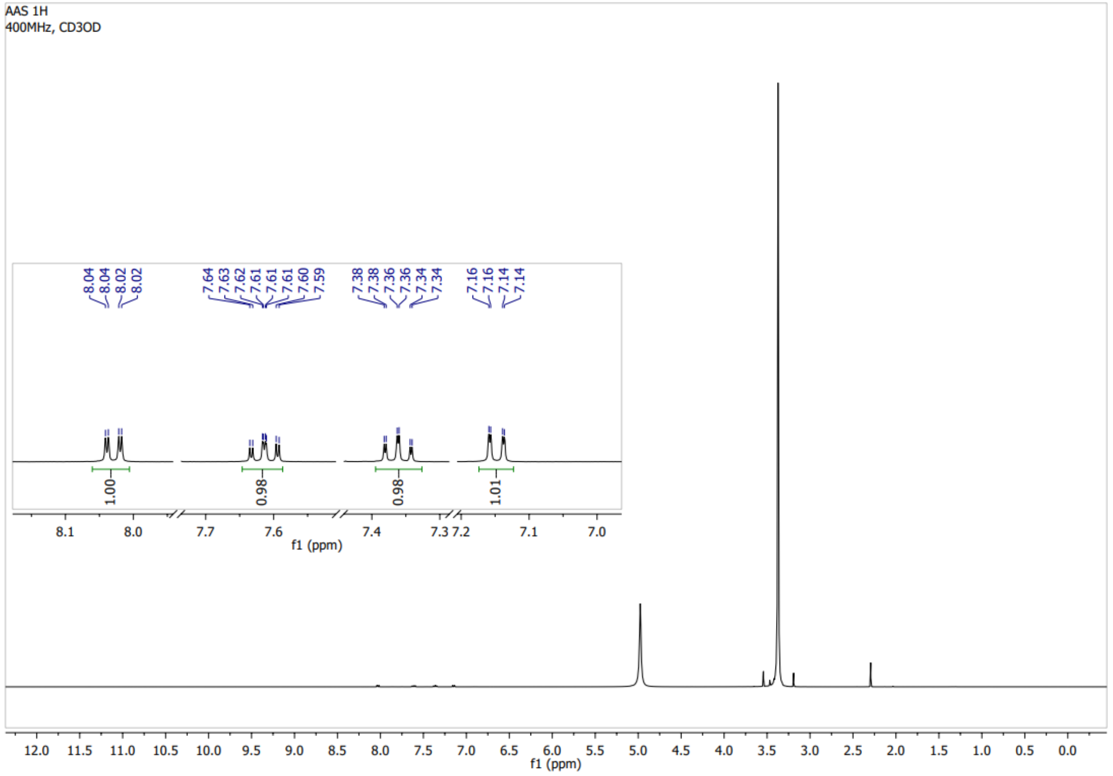

5.1. Figure S8. ^{1}H-NMR [400 MHz, $CDCl_3$, δ (ppm)] of Acetylsalicylic acid (ASA): 2.30, s, 3H (CH_3); 8.125, ddd, 1H (H_b) (J_{be} = 8.0 Hz, J_{bd} = 7.1 Hz and J_{bc} = 1.1 Hz); 7.624, dd, 1H (H_c) (J_{cd} = 8.5 Hz, J_{cb} = 1.1

Hz); 7.356, ddd, 1H (H_d) (J_{dc} = 8.5 Hz, J_{db} = 7.1 Hz, J_{de} =1.7 Hz); and 7.142, dd, 1H (H_e) (J_{eb} = 8.0 Hz, J_{ed} = 1.7 Hz).

5.2. Interpretation of ^{1}H NMR Spectrum.

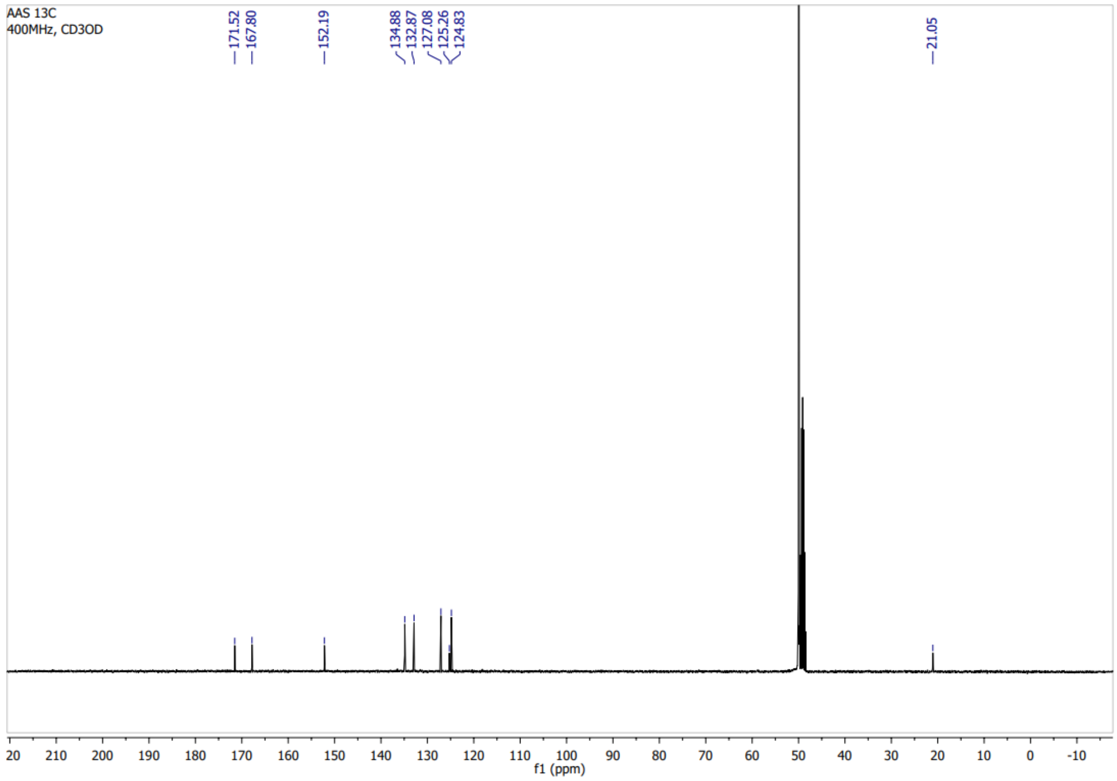

Figure S9. ^{13}C NMR [100 MHz, $CDCl_3$, δ (ppm)] of ASA: 171.5 C=O (CH_3C=O), 167.8 C=O, 152.1 Ar quaternary, 134.8 Ar, 132.8 Ar CH, 127.0 Ar CH, 125.2 Ar CH, 124.8 Ar quaternary, and 49.9 CH_3.

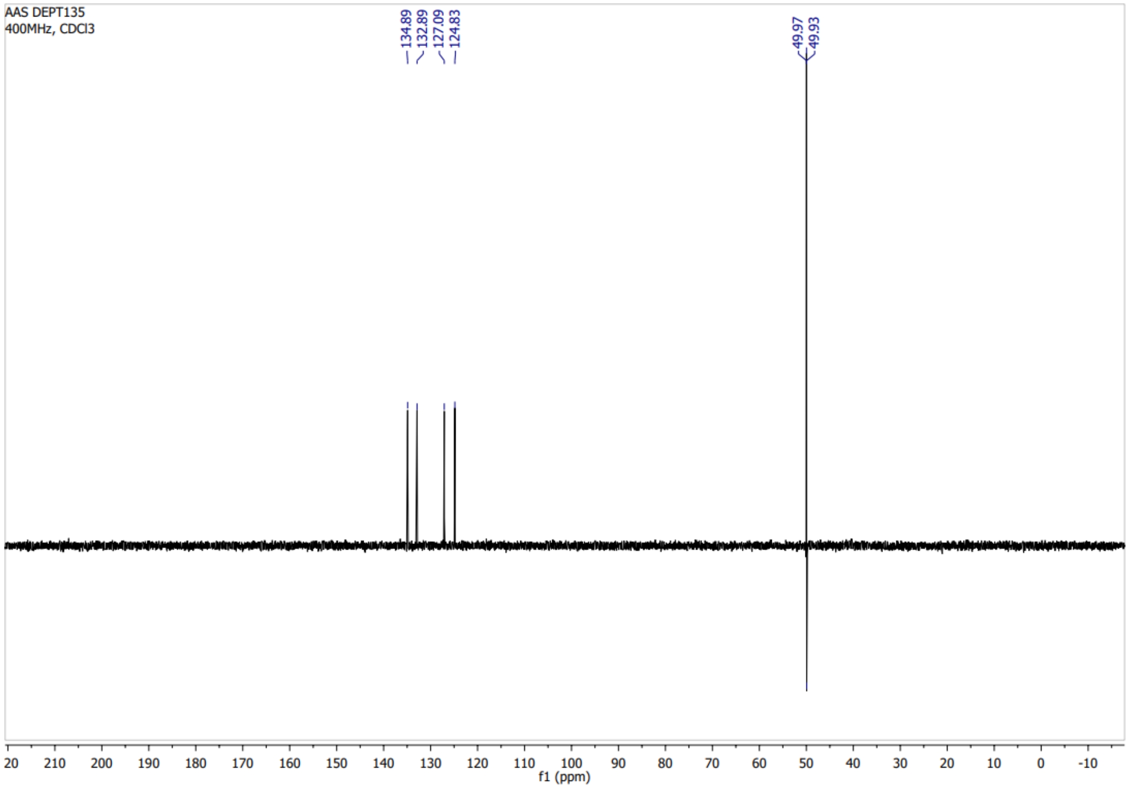

Figure S10. DEPT 135 [400 MHz (^{1}H), $CDCl_3$, δ (ppm)] of ASA: 132.8 Ar CH (methine group - tertiary), 127.0 Ar CH, 125.2 Ar CH, 124.8 Ar CH, and 49.9 CH_3.
